# Room-temperature electrically pumped near-infrared random lasing from high-quality m-plane ZnO-based metal-insulator-semiconductor devices

**DOI:** 10.1186/s11671-015-0816-4

**Published:** 2015-03-01

**Authors:** Chao Chen, Ti Wang, Hao Wu, He Zheng, Jianbo Wang, Yang Xu, Chang Liu

**Affiliations:** Key Laboratory of Artificial Micro- and Nano-structures of Ministry of Education, School of Physics and Technology, Wuhan University, Wuhan, 430072 People’s Republic of China; Center for Electron Microscopy, School of Physics and Technology, Wuhan University, Wuhan, 430072 People’s Republic of China

**Keywords:** m-plane ZnO, MIS, Random lasing

## Abstract

Epitaxial m-plane ZnO thin films have been deposited on m-plane sapphire substrates at a low temperature of 200°C by atomic layer deposition. A 90° in-plane rotation is observed between the m-plane ZnO thin films and the sapphire substrates. Moreover, the residual strain along the ZnO [−12-10] direction is released. To fabricate metal-insulator-semiconductor devices, a 50-nm Al_2_O_3_ thin film is deposited on the m-plane ZnO thin films. It is interesting to observe the near-infrared random lasing from the metal-insulator-semiconductor devices.

## Background

Due to its wide direct bandgap and large exciton binding energy (60 meV), ZnO is an attractive material for optoelectronics, such as ultraviolet (UV) light-emitting diodes [1-3], photodetectors [4], and lasers [5]. Because of the lowest surface free energy of the wurtzite structure {0001} plane, ZnO thin films tend to grow along the <0001 > direction in most cases. Note that the wurtzite structure is polarized along the c-axis caused by spontaneous and piezoelectric polarizations, resulting in the quantum-confined Stark effect and decrease of the internal quantum efficiency of light-emitting devices. Therefore, semi-polar and nonpolar structures are proposed to diminish or even eliminate the polarization effects, and the nonpolar m-plane is one of the common structures [6]. Unfortunately, due to the coexistence of minor domains with <0001>, <11-22>, or <10-13 > growth directions in m-plane ZnO thin films [7-9], it is difficult to deposit high-quality and smooth m-plane ZnO thin films by several normal growth techniques, such as molecular beam epitaxy [7,8], metalorganic chemical vapor deposition [9], and pulsed laser deposition [10]. In addition, with different growth conditions, various striped features are usually presented on the surface of m-plane ZnO thin films deposited on foreign substrates [11,12]. These disadvantages limit the applicability of m-plane ZnO thin films. Furthermore, epitaxial growth of single-crystal ZnO thin films always needs a very high growth temperature. The higher growth temperature may cause defects such as oxygen vacancies in epitaxial films. Featured by layer-by-layer growth, atomic layer deposition (ALD) is one of the most promising techniques that possess several practical advantages including accurate thickness control, good reproducibility, excellent conformity, capability to produce sharp interfaces, and low growth temperature. Especially, among these advantages, low growth temperature is the most attractive one, which makes this technology possible on soft substrates. Although several works have presented epitaxial m-plane ZnO thin films on m-plane sapphire substrates by ALD, the optoelectronic applications of this material still remain an open topic [13,14].

Due to the potential applications in photonics, random lasing has received considerable attention, and ZnO is ideally suited for random lasing because of its high refractive index. Many researchers have already reported random lasing action in ZnO powder and polycrystalline thin films [15-18]. However, almost all of the devices exhibit UV random lasing, and it is seldom reported that a material possesses near-infrared (NIR) random lasing. With these investigations, it is undoubted that electrically pumped ZnO random lasing has promising applications.

Here, m-plane ZnO thin films have been deposited on m-plane sapphire substrates by ALD at 200°C. X-ray diffraction (XRD) and transmission electron microscopy (TEM) results show that high-crystalline-quality m-plane ZnO thin films can be fabricated at such a low growth temperature. In addition, a 50-nm Al_2_O_3_ thin film is deposited on the top of the m-plane ZnO thin films by ALD to fabricate metal-insulator-semiconductor (MIS) devices, and NIR random lasing is observed from the MIS devices.

## Methods

ZnO thin films were grown on m-plane sapphire substrates at 200°C by ALD (Beneq TSF-200, Beneq Oy, Espoo, Finland) with diethyl zinc (DEZn) and deionized water (H_2_O) as precursors. The detailed experimental processes can be found in our previous work [19,20]. The thickness of the ZnO films was about 600 nm. Afterwards, a 50-nm Al_2_O_3_ thin film was deposited on the ZnO thin films at 200°C. Trimethyl aluminum and H_2_O were used as the aluminum and oxygen sources, respectively [21]. A 50-nm-thick Au thin film was deposited on the Al_2_O_3_ thin film as the electrode, and In was used as the electrode for the ZnO thin film.

XRD (Bede D1, Bede Scientific Instruments, Durham, England, UK), TEM (JEOL JEM2010, JEOL Ltd., Akishima-shi, Tokyo, Japan), and high-resolution transmission electron microscopy (HRTEM; JEOL JEM2010FEF, JEOL Ltd., Akishima-shi, Tokyo, Japan) were used to evaluate the crystallization, microstructure, and interface properties. The surface morphology and roughness of the ZnO thin films were characterized by atomic force microscopy (AFM; SHIMADZU SPM-9500 J3, SHIMADZU Corporation, Nakagyo-ku, Kyoto, Japan). The chemical compositions and the defect states of the ZnO thin films are investigated by X-ray photoelectron spectroscopy (XPS; Thermo Scientific ESCALAB 250Xi, Thermo Fisher Scientific, Waltham, MA, USA). Photoluminescence (PL; HORIBA LabRAM HR800, HORIBA, Ltd., Minami-ku, Kyoto, Japan) measurements were conducted at room temperature to analyze the optical properties of the ZnO thin films in a wavelength range of 350 to 700 nm. Electroluminescence (EL) measurements were carried out at room temperature to assess the performance of the MIS devices.

## Results and discussion

**Figure 1:**
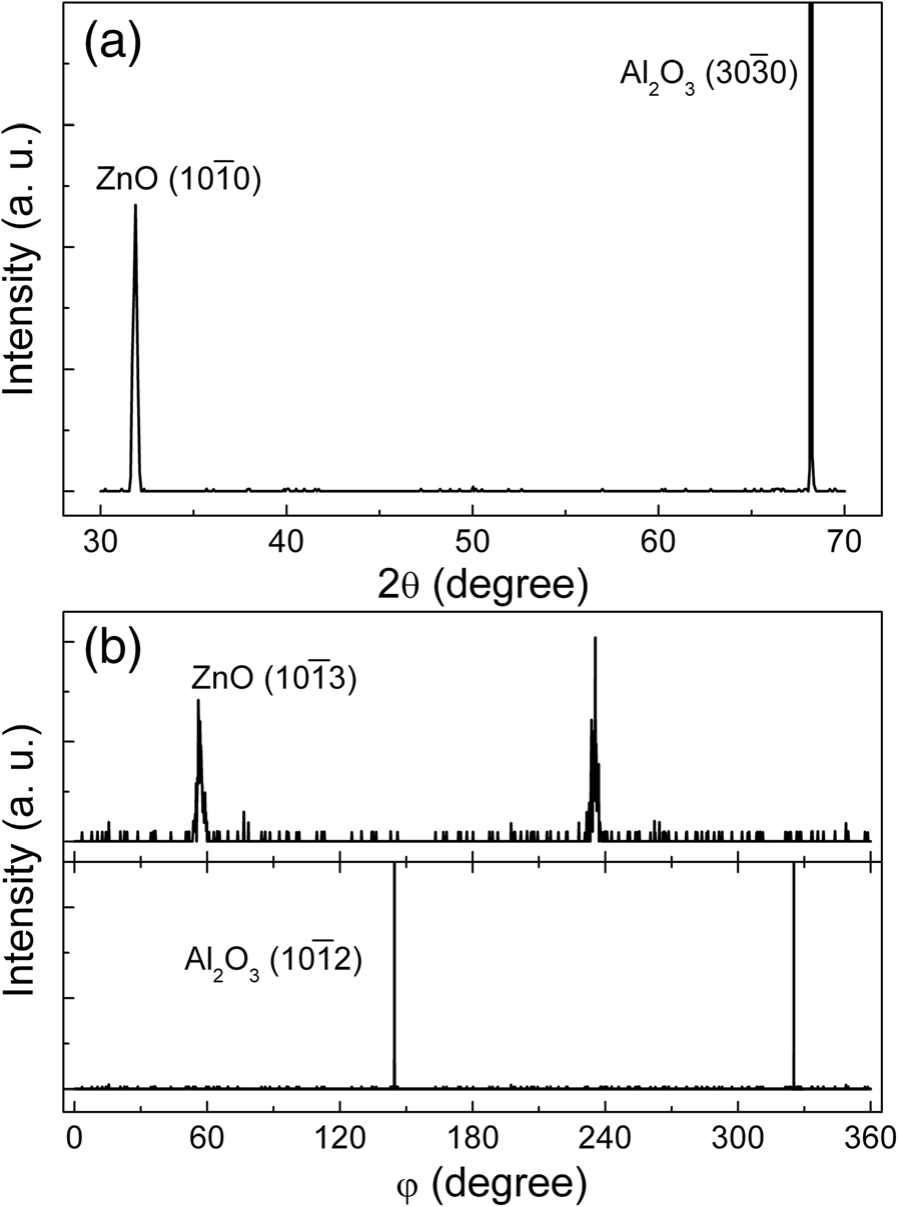
**XRD scans of the ZnO thin films grown on m-plane sapphire. (a)** XRD spectra of the ZnO thin films grown on m-plane sapphire substrates. **(b)** XRD φ scans of sapphire (10–12) and ZnO (10–13).

Figure 1a shows the XRD spectra of the ZnO thin films deposited on the m-plane sapphire substrates, in which the ZnO (10–10) peak is observed besides the sapphire (30–30) peak, clearly indicating that the ZnO thin films are predominantly grown along the [10] direction. The φ scans of the sapphire (10–12) peak and ZnO (10–13) peak are presented in Figure 1b. Both of them show a binary symmetry, revealing that the ZnO thin films are epitaxial on the sapphire substrates. An angle of 90° between ZnO (10–13) peaks and sapphire (10–12) peaks in φ scan spectra is noted. Therefore, it can be deduced that there exists a 90° rotation between the m-plane ZnO thin films and m-plane sapphire substrates. The in-plane orientations are ZnO [0001]//sapphire [1–210] and ZnO [−12-10]//sapphire [0001].

**Figure 2:**
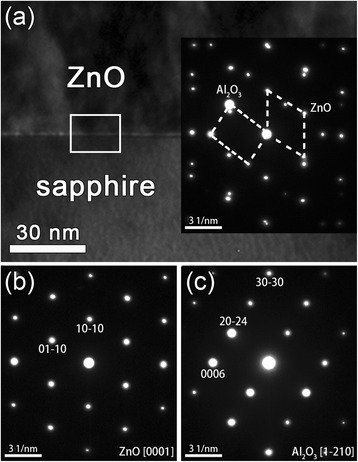
**Bright-field images and SAED patterns. (a)** Bright-field images of the m-plane ZnO on m-plane sapphire. The inset of **(a)** shows the SAED pattern at the interface. **(b)** SAED pattern obtained from the ZnO thin film. **(c)** SAED pattern obtained from the m-plane sapphire substrate.

**Figure 3:**
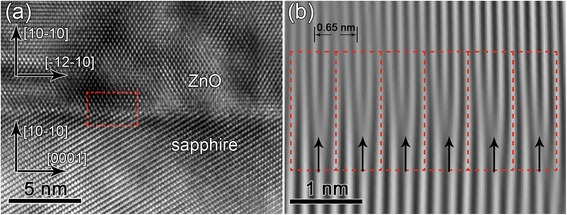
**Cross-sectional HRTEM and IFFT-filtered images. (a)** Cross-sectional HRTEM image of the interface (the magnified image of the boxed area in Figure 2a). **(b)** IFFT-filtered image of the red dashed box of **(a)** using ZnO (−12-10) and (1–210) reflections and sapphire (0006) and (000–6) reflections.

To further analyze the crystalline quality and the in-plane orientation, TEM studies were performed. As is shown in Figure 2, the ZnO thin film deposited by ALD has a very sharp interface. Figure 2b,c and the inset of Figure 2a show the selected area electron diffraction (SAED) patterns of the sapphire substrate, the ZnO thin film, and the interface region, respectively. The growth direction of the ZnO thin film is along [10] as presented in Figure 2b, in full agreement with the XRD result. The orientation relationships between the ZnO thin film and the sapphire substrate can be manifested as follows: ZnO [0001]//sapphire [1–210] and ZnO (−12-10)//sapphire (0006), which are demonstrated in the inset of Figure 2a. Furthermore, it is found that the ZnO thin film epitaxially grows on the m-plane sapphire substrate and has a high crystalline quality as demonstrated by the HRTEM image shown in Figure 3a. The HRTEM image is taken along ZnO [0001], and the epitaxial relationship between ZnO and sapphire is ZnO [10]//sapphire [10] and ZnO [−12-10]//sapphire [0001], consistent with the SAED results. The interface is observed to be atomically sharp. The mismatch-induced strains are usually relaxed through the generation of misfit dislocations (MDs) at the interface in heteroepitaxial structures. Figure 3b shows the magnified image of the white boxed area in Figure 2a. Figure 3b presents the inverse fast Fourier transform (IFFT)-filtered image of the red dashed area in Figure 3a by using ZnO (−12-10) and (1–210) reflections and sapphire (0006) and (000–6) reflections. The MDs are clearly visible in Figure 3b, and extra half-planes within the sapphire substrate are marked by the arrows. In each dashed boxed area, an extra half-plane is showed and the lattice fringes of ZnO and sapphire present 4:3. Considering the interplanar distances of ZnO (−12-10) (0.1625 nm) and sapphire (0006) (0.2165 nm), the lattice misfit (*δ*) along the ZnO [−12-10] direction is calculated to be 24.94%. However, with four ZnO (−12-10) planes and three sapphire (0006) planes, it can be matched nearly perfectly with a mismatch only about 0.08%. In this way, the residual strain along the ZnO [−12-10] direction is released. Moreover, the equilibrium spacing (*D*) is calculated to be 0.6516 nm via the following formula: *D* = |*b*|/*δ*, where |*b*| is the magnitude of the Burgers vector component parallel to the interface, i.e., 0.1625 nm $\left(={d}_{\mathrm{ZnO}}^{\hbox{-} 12\hbox{-} 10}\right)$. The calculated equilibrium spacing of the MDs is close to the observed average spacing between the MDs marked by arrows in Figure 3b (i.e., 0.65 nm).

**Figure 4:**
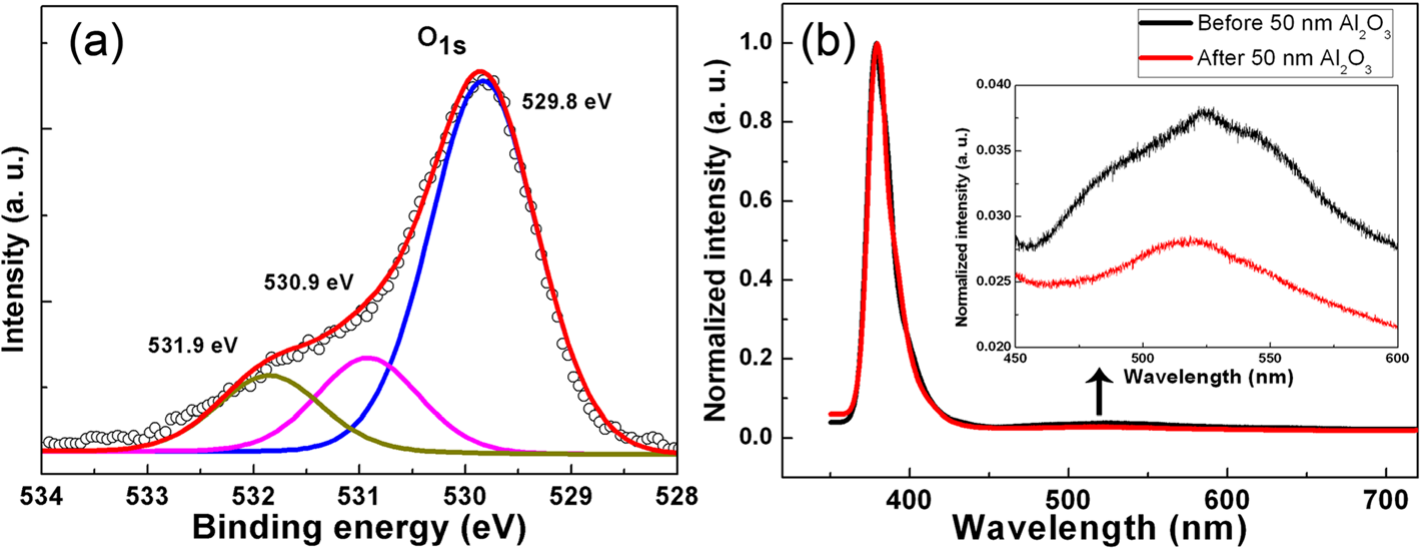
**O 1** ***s*** **XPS spectra and PL spectra of the ZnO thin films. (a)** O 1 *s* XPS spectra of the ZnO thin films. Dots are the experimental data and lines are the fitting results. **(b)** The PL spectra of the ZnO thin films before and after depositing a 50-nm Al_2_O_3_ cap layer. The inset of **(b)** is the magnified area of the PL spectra from 450 to 600 nm.

In order to analyze the chemical compositions and the defect states of the ZnO thin films, XPS measurement was performed. From the statistical results of XPS, the atom ratio of Zn:O is about 1.06:1, in which it can be observed that the ZnO thin films are a little Zn-rich. Figure 4a shows the O 1 *s* XPS spectra of the ZnO films. The binding energies of the XPS spectra have been calibrated by taking the carbon C 1 *s* peak (284.5 eV) as a reference. The O 1 *s* peak is deconvoluted into three peaks at 529.8, 530.9, and 531.9 eV by using Gaussian fitting. The peaks centered at 529.8 and 530.9 eV are assigned to oxygen atoms in the oxide lattice without and with oxygen vacancies, respectively. The peak at 531.9 eV is usually attributed to chemisorbed oxygen on the surface of ZnO thin films, such as carbonyl and hydroxyl groups. The relative integrated intensity percentages of the different oxygen species calculated from fitting peaks are used to analyze the quantities of the oxygen lattice, oxygen vacancy, and hydroxyl group [22,23]. The percentages of peaks centered at 529.8, 530.9, and 531.9 eV are 69.53%, 16.68%, and 13.8%, respectively. Compared with the XPS results from other works [23,24], our ZnO films have a high percentage of the oxygen lattice-related peak and a small ratio of the oxygen vacancy-related peak. Therefore, from the XPS results, it can be concluded that there are only a few oxygen vacancies in the ZnO thin films deposited at 200°C by ALD.

To fabricate MIS devices, a 50-nm Al_2_O_3_ thin film was deposited on the m-plane ZnO thin film by ALD. Figure 4b shows the PL spectra of the ZnO thin films with and without the Al_2_O_3_ thin film. The intensities of the peaks have been normalized. Both spectra display a dominant sharp near-band-edge (NBE) emission and a weak deep-level emission around 520 nm. The deep-level emission has been reported to be caused by oxygen vacancies. Hence, it indicates that few oxygen vacancies exist in the ZnO thin films, which is consistent with the XPS results. Note that they show similar UV emissions though the deep-level emission has decreased after depositing the Al_2_O_3_ thin film as shown in the inset of Figure 4b. The only difference between the two samples is with or without the 50-nm Al_2_O_3_ thin film. Therefore, the decrease of the deep-level emission intensity can be attributed to the decrease of oxygen vacancies at the interface between ZnO and Al_2_O_3_.

**Figure 5:**
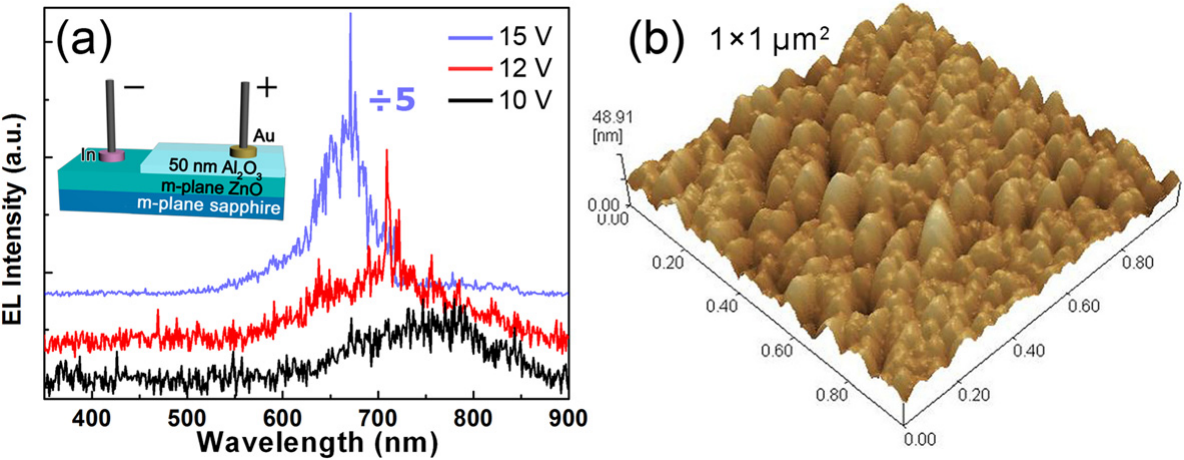
**EL spectra of the MIS devices and AFM image of the ZnO thin film. (a)** EL spectra of the MIS devices. The inset of **(a)** is the schematic diagram of the MIS devices. **(b)** AFM image of the ZnO thin film.

The inset of Figure 5a shows the schematic diagram of the m-plane ZnO-based MIS devices. Au and In were used as electrodes on the Al_2_O_3_ thin films and the ZnO thin films, respectively. Figure 5a presents the EL spectra of the MIS devices under various forward biases. NIR emission peaks are observed, whereas no UV emission peaks are found in the EL spectra. As is known, the MIS structures can increase carrier density in the radiative recombination region by confining carriers in the semiconductor-insulator interface. Consequently, the main recombination occurred at the interface in MIS devices. Wang et al. have observed NIR emissions from the PL spectra of ZnO microspheres and attributed the NIR emissions to oxygen interstitials as deep acceptors [25]. With the 50-nm Al_2_O_3_ thin film, the amount of oxygen vacancies reduces at the interface demonstrated by PL measurements. Therefore, due to the 50-nm Al_2_O_3_ cap layer, the surface of the m-plane ZnO thin film has been changed and oxygen interstitials emerged at the interface. As a result, NIR emissions are generated due to the radiative recombinations between shallowly trapped electrons and the deeply trapped holes at oxygen interstitials [25]. In addition, it is interesting that NIR random lasing has been observed above the forward bias of 12 V, which appears as randomly distributed and intensive sharp peaks in the EL spectra. When the bias increases, more electrons accumulate at the interface between ZnO and Al_2_O_3_, which leads to more transitions directly from the conduction band edge to the oxygen interstitial level. As a consequence, photons with higher energy are emitted from the luminescent region, and the EL emission center is blueshifting with the increase of the bias. The generating mechanism of NIR random lasing needs to be further systematically studied. However, a similar random lasing has been observed in some works, and they deduced that the random lasing phenomenon was caused by light scattering in the luminescent region. The origin of the scattering can be attributed to total internal reflection at grain boundaries [5], fluctuations of refractive indexes at the interface of heterostructures [26], refractions among the grains [15], etc. To confirm the possibility of this mechanism in our system, the surface morphology of the ZnO thin films was characterized by AFM measurement. As is shown in Figure 5b, the surface is smooth and uniform, with a root-mean-square roughness of only 5.7 nm. But it presents that there are many grains at the surface and the size of the grains is generally below 100 nm. These grains and grain boundaries can be the reasons for light scattering. As a consequence, through multiple scattering, light may return to the scatterer from which it has been scattered before and results in the self-formation of a closed loop path. The loop path with coherent feedback of the scattering light can achieve optical gain, and thus, it serves as a resonator for that light frequency. Distinguished from the other light-emitting devices, radiative transitions in the MIS devices are confined at the interface between the semiconductor and insulator. Such concentrated luminescence can greatly increase the chance to form closed loop paths. Due to the randomness of the formation of closed loop paths, their respective resonance frequencies result in discrete sharp random peaks in the emission spectrum.

## Conclusions

In summary, high-crystalline-quality m-plane ZnO thin films have been deposited on m-plane sapphire substrates with a low growth temperature. XRD results denote that the in-plane orientations are ZnO [0001]//sapphire [1–210] and ZnO [−12-10]//sapphire [0001], which can also be demonstrated by SAED images. It can be speculated from the HRTEM results that the residual strain along the ZnO [−12-10] direction was nearly zero. Moreover, to fabricate MIS devices, a 50-nm Al_2_O_3_ thin film was deposited on the m-plane ZnO thin films by ALD, and it was interesting to observe NIR random lasing from the MIS devices.
